# Lipid droplet dynamics during *Schizosaccharomyces pombe* sporulation and their role in spore survival

**DOI:** 10.1242/bio.022384

**Published:** 2017-01-03

**Authors:** Hui-Ju Yang, Hiroko Osakada, Tomoko Kojidani, Tokuko Haraguchi, Yasushi Hiraoka

**Affiliations:** 1 Graduate School of Frontier Biosciences, Osaka University, Suita, Japan; 2 Advance ICT Research Institute Kobe, National Institute of Information and Communications Technology, Kobe, Japan; 3 Japan Women's University, Tokyo, Japan

**Keywords:** Actin, Forespore membrane, Lipid droplet, Septation initiation network, Spore, Germination

## Abstract

Upon nitrogen starvation, the fission yeast *Schizosaccharomyces pombe* forms dormant spores; however, the mechanisms by which a spore sustains life without access to exogenous nutrients remain unclear. Lipid droplets are reservoirs of neutral lipids that act as important cellular energy resources. Using live-cell imaging analysis, we found that the lipid droplets of mother cells redistribute to their nascent spores. Notably, this process was actin polymerization-dependent and facilitated by the leading edge proteins of the forespore membrane. Spores lacking triacylglycerol synthesis, which is essential for lipid droplet formation, failed to germinate. Our results suggest that the lipid droplets are important for the sustenance of life in spores.

## Abstract (precis)

**Summary:** Lipid droplets of yeast mother cells are shown to redistribute to their nascent spores by live-cell imaging analysis, suggesting that the lipid droplets are important for yeast spore survival.

## INTRODUCTION

Spore formation represents a fungal survival strategy under unfavorable conditions. Lipid droplets (LDs) are implicated in fungal spore development (Fan et al., 2015; Lin et al., 2013; Ren et al., 2014); however, the precise role of LDs in spore development remains elusive. An LD is a membrane monolayer organelle that is primarily comprised of the neutral lipids triacylglycerols (TAGs) and sterol esters (Thiam et al., 2013). LDs play a role in diverse biological pathways involved in the supply of lipids for membrane synthesis, energy production, and formation of lipophilic molecules (Blom et al., 2011; Dichlberger et al., 2013; Pol et al., 2014; Rambold et al., 2015; Shpilka et al., 2015), and interact with various other organelles to exert specific functions (Gao and Goodman, 2014). To elucidate the role of LDs in spore development, an understanding of the dynamic movements of these organelles during sporulation is required.

The fission yeast *Schizosaccharomyces pombe* undergoes sporulation when deprived of nitrogen sources. Upon induction of sporulation, the yeast enters meiosis to generate four haploid nuclei in an ascus. These haploid nuclei are packaged into four ascospores capable of survival under nutrient-limited conditions. Spore packaging begins with the assembly of the forespore membrane (FSM), which will subsequently be utilized as the spore plasma membrane, and is assembled via fusion of the membrane vesicles at the spindle pole body (SPB) during meiosis II (Ikemoto et al., 2000; Nakase et al., 2008). A proportion of the membrane vesicles arise from robust endocytosis of the ascus plasma membrane (Kashiwazaki et al., 2011). The endocytic membrane vesicles transport cargo, including the SNARE protein Psy1, to the meiotic SPB (Nakamura et al., 2008, 2001). Vesicle tethering at the SPB is facilitated by the Rab GDP/GTP exchange factor Spo13 localized at the cytoplasmic plaque of the meiotic SPB (Yang and Neiman, 2010), and these vesicles subsequently fuse with each other to form the FSM through SNARE complex formation (Maeda et al., 2009; Nakamura et al., 2005; Neiman, 1998; Yang et al., 2008).

The opening of a growing FSM is decorated with the leading edge proteins (LEPs), which assemble into ring structures at the leading edge and guide the FSM along the nuclear envelope (Moreno-Borchart et al., 2001; Neiman, 2011). In *S. pombe*, the LEP rings are comprised of Meu14, actin, and Mcp4 (Ohtaka et al., 2007; Okuzaki et al., 2003; Yan and Balasubramanian, 2012). Following capture of the nucleus by the FSM, constriction of the LEP rings facilitates FSM closure (Diamond et al., 2008; Yan and Balasubramanian, 2012).

FSM closure is a process equivalent to cytokinesis, separating the ascus cytoplasm from the spore cytoplasm. The septation initiation network (SIN), which regulates cytokinesis, modulates sporulation in *S. pombe* (Goyal et al., 2011; Krapp et al., 2006), and a kinase cascade that occurs during SIN signaling ultimately activates the nuclear Dbf2-related (NDR) kinases (Rhind and Russell, 2012). Notably, a strain harboring a deletion of the gene encoding the meiosis-specific NDR kinase Mug27 (*mug27*Δ) produced FSMs that were small in size and frequently failed to enclose the nucleus during spore formation (Ohtaka et al., 2008; Perez-Hidalgo et al., 2008; Yan et al., 2008). Moreover, meiotic actin ring constriction in NDR-kinase mutants show slow kinetics (Yan and Balasubramanian, 2012), indicating that SIN signaling regulates FSM closure.

In this study, we examined the dynamics of LDs in sporulating cells of *S. pombe*. LDs were actively transported to forespores, and most LD-depleted spores were incapable of germination.

## RESULTS AND DISCUSSION

### LDs form clusters during meiosis II and partition into forespores

**Fig. 1.**
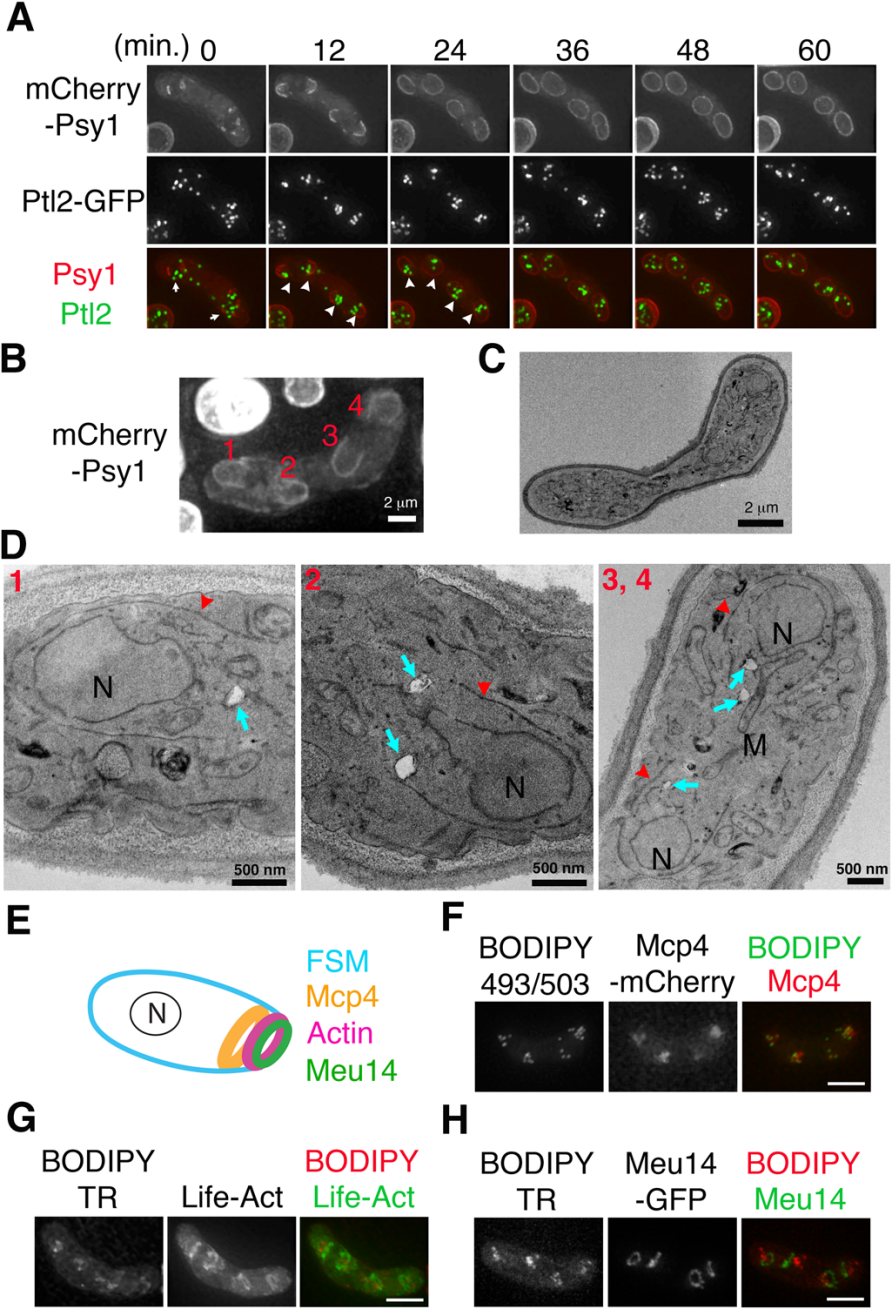
**Dynamics of LDs during spore formation.** (A) Representative time-lapse images of the FSMs engulfing the LDs observed in living *S. pombe* cells expressing the FSM marker mCherry-Psy1 and the LD marker Ptl2-GFP (11 cells observed). FSM and LDs are shown in red and green, respectively. LDs appeared as distinct focal structures. FSM initiation (detected as aggregation of the mCherry-Psy1 fluorescence signals in the cytoplasm) was designated as 0 min. The arrows at 0 min indicate clustering of LDs near the FSM initiation site. The arrowheads at 12 min indicate four LD clusters near the FSM leading edges. The arrowheads at 24 min indicate inclusion of the LDs by FSM extension. Scale bar: 5 µm. (B) Fluorescence images of FSM extension. Cells expressing mCherry-Psy1 were fixed for EM imaging (see Materials and Methods). The numbers 1–4 represent the four FSMs. Scale bar: 2 µm. (C) TEM image of the same cell shown in (B). Scale bar: 2 µm. (D) Magnified TEM images of the cell depicted in (C). Each of the numbered images corresponds to the numbered FSMs in (B). The red arrowheads indicate the FSM. The cyan arrows indicate LDs, which appear as white matter when visualized by TEM. N, nucleus; M, mitochondria. Scale bar: 500 nm. (E) Localization of the LEP rings at the FSM leading edge. N, nucleus. (F-H) Co-localization of LDs and LEPs. Immediately before imaging, the fluorescent dye BODIPY493/503 or BODIPY TR was added to the sporulation medium containing cells expressing Mcp4-mCherry, Meu14-GFP, or LifeAct-GFP. The BODIPY dyes stain LDs. LifeAct-GFP binds to actin filaments to allow visualization of the meiotic actin ring (Yan and Balasubramanian, 2012). Scale bar: 5 µm.

To elucidate the mechanism by which spores acquire LDs, we observed living sporulating cells expressing Ptl2-GFP. Ptl2, a TAG lipase of *S. pombe* (Yazawa et al., 2012), localizes to the LDs (Fig. S1); the average number of LDs labeled by Ptl2-GFP in a sporulating cell was 25. LDs showed dynamic movements during sporulation, scattering in the cytoplasm during meiosis I (Fig. S2, 0–18 min), but clustering around the two divided nuclei just before the onset of meiosis II (Fig. S2, 36–42 min). Clustering of LDs occurred in proximity to the site of initiation of FSM assembly in meiosis II (Fig. 1A, arrows). However, in the *spo13*Δ mutant, LDs clustered efficiently at the nucleus without FSM assembly (Fig. S3), indicating that LD clustering occurs independent of FSM assembly. As the FSM grew into a crescent-shaped structure in anaphase II, the LD clusters further partitioned into each of the four FSMs (Fig. 1A, 12–24 min, arrowheads). Continuous extension of the FSM eventually enclosed the LDs within the forespore (Fig. 1A, 24–60 min).

LDs were found in close proximity to the leading edges of the FSM during FSM extension (Fig. 1A, 12 min, arrowheads). Consistently, when the cell undergoing FSM extension was further subjected to electron microscopy (EM) (Fig. 1B,C), LDs were often observed near the leading edge of each FSM (Fig. 1D, arrows). FSM leading edges are decorated by the three LEP rings: the Meu14 ring located at the ascus cytoplasmic side of the FSM leading edge; the Mcp4 ring at the future spore cytoplasmic side; and the meiotic actin ring situated between the Meu14 ring and the Mcp4 ring (Fig. 1E) (Ohtaka et al., 2007). Co-localization analysis revealed that LDs closely associate with the Mcp4 ring, but localize behind the meiotic actin ring and the Meu14 ring (Fig. 1F–H), indicating that the LDs were located at the future spore cytoplasm.

### LEPs facilitate efficient inclusion of LDs by the FSM

**Fig. 2.**
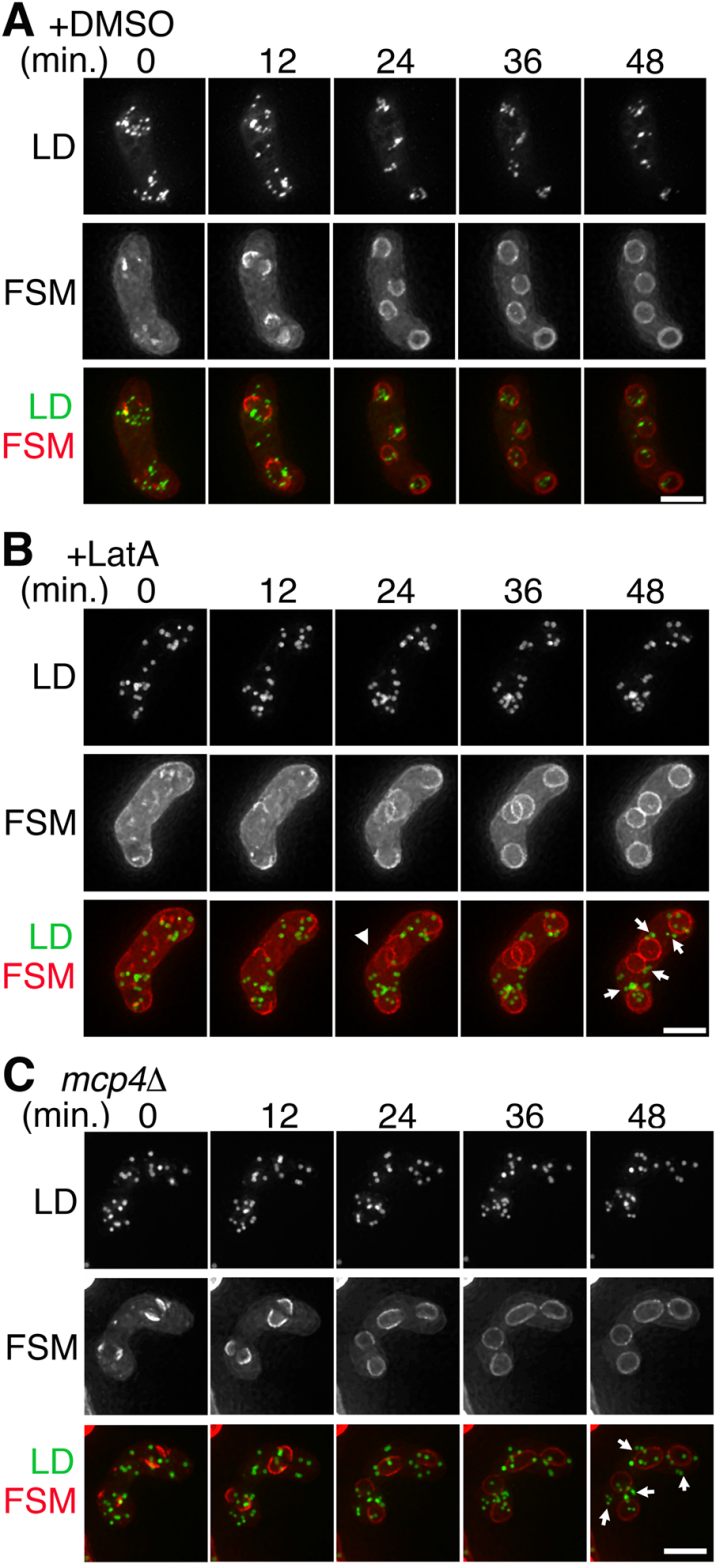
**Actin polymerization is required for LD clustering.** Time-lapse images of living cells expressing mCherry-Psy1 (red) and Ptl2-GFP (green): wild-type cell treated with DMSO (A), wild-type cell treated with Latrunculin A (final concentration of 1 µM) (B), and the *mcp4*Δ mutant (C). The arrowheads at 24 min indicate the FSM with few associated LDs. The arrows at 48 min represent LDs in the exterior of the forespores. The presented image is a representative example: the number of cells observed is 10 for (A), 12 for (B) and 15 for (C). Scale bar: 5 µm.

We next examined whether the LEPs play a role in LD movement. The meiotic actin ring was dissembled by treating the sporulating cells with the actin polymerization inhibitor Latrunculin A. While most LDs clustered at the FSM initiation sites in the control cells (Fig. 2A, 0 min), the LDs in the Latrunculin A-treated cells remained scattered upon initiation of FSM assembly (Fig. 2B, 0 min), suggesting that actin polymerization is required for LD clustering at the FSM assembly site. Furthermore, the FSM leading edge in the Latrunculin A-treated cells was associated with few or no LDs (Fig. 2B, 24 min, arrowheads), resulting in inefficient inclusion of LDs by FSMs in these cells (Fig. 2B, 48 min, arrows). As in the Latrunculin A-treated cells, LDs failed to cluster well at the FSM initiation site, and numerous LDs were excluded from the spore cytoplasm in the *mcp4*Δ mutant (Fig. 2C, 48 min, arrows). The similarity in the phenotype of Latrunculin A-treated cells and *mcp4*Δ cells is consistent with a previous study reporting that Mcp4 is involved in F-actin positioning (Ohtaka et al., 2007).

**Fig. 3.**
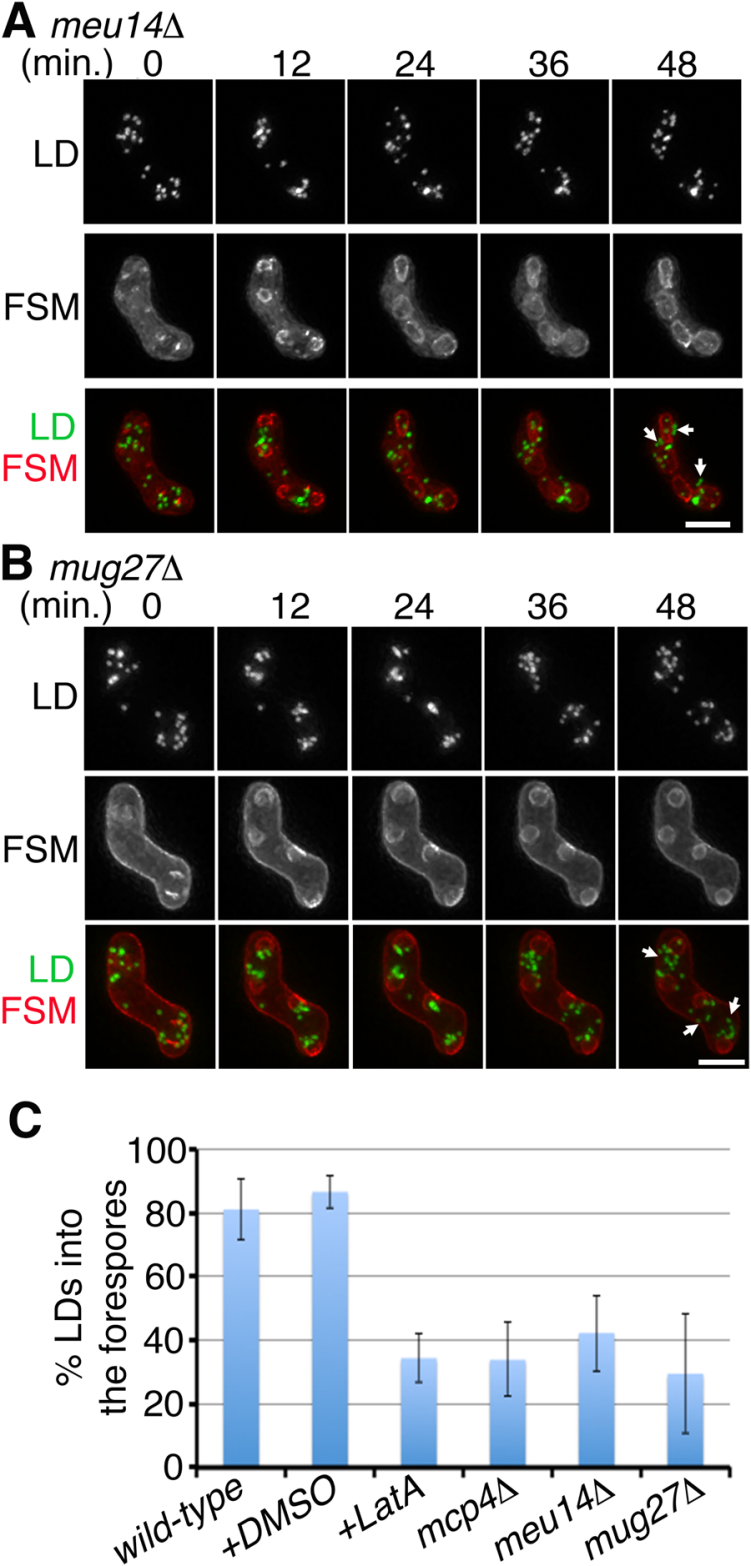
**LEPs are important for efficient inclusion of LDs.** (A,B) Representative time-lapse images of LD movements during FSM assembly in the *meu14*Δ mutant (A) (12 cells observed) and the *mug27*Δ mutant (B) (14 cells observed); Ptl2-GFP (LDs) and mCherry-Psy1 (FSMs) are shown in green and red, respectively. The arrowheads at 12 min indicate the FSM leading edges, without association of the LDs in the *meu14*Δ mutant. The arrows at 48 min indicate the LDs remaining in the ascus cytoplasm. Scale bar: 5 µm. (C) Quantification of LDs enclosed by FSMs in the various strains. Time-lapse images of 10 samples for each strain were counted. The number of LDs in a sporulating cell was quantified at 48 min or after. Percentage of LDs transported into the forespore=(number of LDs enclosed by the FSM/total number of LDs)×100%. The graph and the error bar represent mean and standard deviation, respectively.

Meanwhile, depletion of the Meu14 ring had little effect on initial clustering of LDs (Fig. 3A, 0 min). In contrast with the wild-type cells (Fig. 1A, 12 min, arrowheads), the *meu14*Δ mutant exhibited poor association of LD clusters with the FSM leading edges (Fig. 3A, 12 min, arrowheads). As a result, LDs were not enclosed by the FSM, instead remaining in the ascus cytoplasm in the *meu14*Δ mutant (Fig. 3A, 48 min, arrows).

We propose that LD transport into spores involves two steps: first, actin polymerization is required for LD clustering at the FSM assembly site; second, the LEP rings facilitate efficient inclusion of LDs by the FSM. A previous study demonstrated that Mug27 regulates constriction of the meiotic actin rings without affecting their assembly (Yan and Balasubramanian, 2012). Accordingly, we examined LD movements in the *mug27*Δ mutant. In agreement with our hypothesis, initial clustering of LDs was normal in the *mug27*Δ mutant (Fig. 3B, 0 min); however, most of the LDs were still excluded from the forespores (Fig. 3B, 48 min, arrows). Therefore, fewer LDs were enclosed by FSMs in the LEP-disruption mutants than in the wild-type cells (Fig. 3C), indicating that LEP rings mediate LD transport to the forespores.

### LDs are required for spore germination and spore wall integrity

**Fig. 4.**
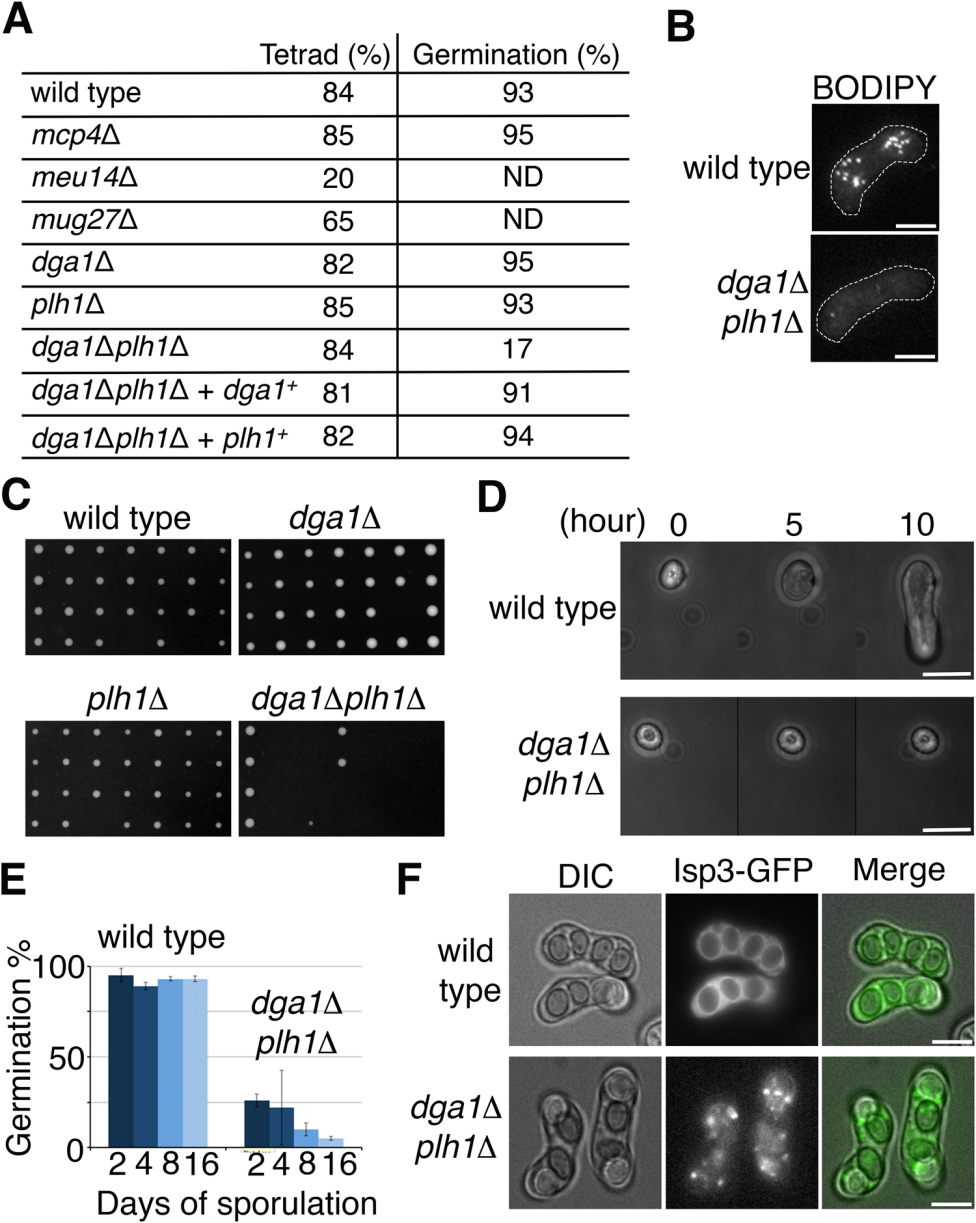
**LDs are important for spore germination and spore wall integrity.** (A) Frequency of tetrad formation and spore germination in the different strains. Tetrad formation frequency=(number of asci with four spores/number of total zygotes)×100%. At least 600 tetrads were scored for each strain. After 3-5 days of sporulation, the rate of spore germination of each strain was determined by assessing spore colony formation through tetrad analysis (42 tetrads were dissected for each strain). Spores that failed to form colonies were further confirmed for germination using a dissecting microscope. Germination frequency=(number of germinated spores/number of total spores)×100%. A *lys1^+^*-integrating plasmid carrying *dga1*^+^ or *plh1*^+^ was used to restore the germination efficiency of the *dga1*Δ*plh1*Δ mutant. (B) The *dga1*Δ*plh1*Δ mutant possessed few LDs. The fluorescent dye BODIPY was used for LD labeling. The white dashed line outlines the sporulating cell Scale bar: 5 µm. (C) Examples of spore colony formation in the *dga1*Δ*plh1*Δ mutant. (D) Representative morphological changes of spore germination over time in the wild-type or the *dga1*Δ*plh1*Δ spore. Scale bar: 6 µm. (E) The germination defect of the *dga1*Δ*plh1*Δ spores was time-dependent. The cells were subjected to sporulation on an ME plate for 2, 4, 8, or 16 days. At each time point, spore germination was assayed by tetrad dissection on the YES plate (three independent experiments per strain; 14 tetrads were dissected per experiment). Germination frequency=(number of germinated spores/number of total spores)×100%. The graph and the error bar represent mean and standard deviation, respectively. (F) The spore wall was improperly assembled in the *dga1*Δ*plh1*Δ mutant. Isp3-GFP was used to visualize the outermost layer of the spore wall. Isp3-GFP fluorescence signals were evenly distributed on the surface of the wild-type ascospores, whereas Isp3-GFP exhibited aggregate formation and uneven decoration of the ascospores of the *dga1*Δ*plh1*Δ mutant. Scale bar: 5 µm.

In addition to enclosing LDs with low efficiency relative to the wild type, the FSMs of *meu14*Δ and *mug27*Δ mutants exhibit abnormal formation and frequently fail to engulf the spore nucleus (Ohtaka et al., 2008; Okuzaki et al., 2003), making it complex to verify the requirement of LDs for spore survival. By contrast, in the present study, the *mcp4*Δ mutant formed four spores per ascus (tetrads) as frequently as wild-type cells (Fig. 4A). We assayed spore survival by analysis of spore germination rate. Fewer than 50% of the LDs were transported into the forespores (Fig. 3C); despite this, the *mcp4*Δ spores germinated well (Fig. 4A). This might be attributable to the incomplete depletion of LDs within the mutant. We therefore deleted the genes required for TAG synthesis. The enzymes Dga1 and Plh1, which convert lipids such as free fatty acids and phospholipids into TAG, are responsible for LD formation in *S. pombe* (Meyers et al., 2016). The characteristic BODIPY-stained punctate structures were largely lost in the sporulating cells of the *dga1*Δ*plh1*Δ mutant, indicating diminishment of the LDs (Fig. 4B). Noticeably, while the *dga1*Δ*plh1*Δ mutant produced a comparable amount of tetrads to the wild-type cells (Fig. 4A), most of the spores (83%) failed to form colonies owing to germination defects (Fig. 4A,C). Those *dga1*Δ*plh1*Δ spores that failed to form colonies showed no sign of germination (Fig. 4D), whereas the wild-type spores exhibited expansion growth, and emergence of germ tubes within 5 to 10 h after transfer to the growth medium, as previously reported (Hatanaka and Shimoda, 2001). The frequency of spore germination was only 17% (Fig. 4A); intriguingly, however, the frequency of tetrads containing four viable spores was strikingly higher (5%; 2 out of 42 asci) than that predicted by random distribution of viable spores in an ascus (0.08%; 0.17^4^=0.0008). This result of non-random distribution indicates that spores in each ascus share the same fate. Thus, it is likely that the viability of spores in the absence of TAG synthesis is metabolically determined during meiosis and sporulation. Although the viability of spores produced by the *dga1*Δ*plh1*Δ mutant largely decreased during sporulation, it further decreased gradually when maintained in the absence of TAG synthesis (Fig. 4E). In contrast, the spores of wild-type cells retained high viability in sporulation medium for 16 days (Fig. 4E). These results indicate that TAG plays a necessary role in spore survival under starvation conditions.

The LD-deficient mutant not only exhibits defects in spore germination but also in spore wall integrity (Fig. 4F). Spore wall deposition after FSM assembly confers resistance to spores against various stresses (Coluccio et al., 2008; Fukunishi et al., 2014). The outermost layer of the *S. pombe* spore wall comprises a protein layer composed of Isp3, which is highly palmitoylated (Fukunishi et al., 2014; Zhang et al., 2013). This Isp3 coating was defective in the spores of the *dga1*Δ*plh1*Δ strain (Fig. 4F), raising the possibility that TAGs mediate the characterized lipid-modification of Isp3. These results indicate that LDs are important for spore germination and spore wall integrity.

LDs are crucial for the survival of starved cells (Rambold et al., 2015; Shpilka et al., 2015). Our study revealed that LDs are actively transported to nascent spores, and that *dga1*Δ*plh1*Δ spores, bearing few LDs, barely germinate. These data indicate that LDs represent an important cellular energy source for spores under starvation conditions. Alternatively, apoptosis may be induced in *dga1*Δ*plh1*Δ spores as a result of their failure to transform diacylglycerol into TAG (Zhang et al., 2003). Further studies will clarify the mechanisms by which LDs support spore survival.

## MATERIALS AND METHODS

### Yeast strains and culture

The *S. pombe* strains used in this study are listed in Table S1. All strains were grown on yeast extract with supplements (YES) plates at 30°C, as described by Moreno et al. (1991). To induce sporulation, freshly cultured cells were collected in nitrogen-free Edinburgh minimal medium (Moreno et al., 1991) supplemented with adenine, uracil, histidine, lysine, and leucine (EMM-N+5S) at a density of 10^9^ cells/ml. Cells were then transferred to malt extract (ME) plates to allow sporulation at 26°C.

Gene disruption was performed using a polymerase chain reaction (PCR)-based strategy (Bähler et al., 1998). The PCR primers used in these analyses are listed in Table S2. For deletion of the *meu14^+^* gene, DNA fragments with homology to the target gene locus were amplified using the primers HJO423, HJO424, HJO425, and HJO426, whereas DNA fragments for the deletion of the *dga1^+^* gene were amplified using the primers HJO684, HJO685, HJO686, and HJO687. The *plh1^+^* gene was replaced with the drug resistance gene module kanMX6 using the plasmid pFA6a-kanMX6 and primers HJO689, HJO690, HJO691, and HJO692. The *mcp4*Δ, *mug27*Δ, and *spo13*Δ strains were derived from strains FY16412, FY17842 and FY12290, respectively (obtained from the Yeast Genetic Resource Center of Japan) (Nakase et al., 2008; Ohtaka et al., 2007, 2008).

To fluorescently label Ptl2, Isp3, or Mcp4, a two-step PCR method introducing the chromosomal GFP or mCherry tag was used (Hayashi et al., 2009). To visualize the FSM, integrating plasmids carrying mCherry-*psy1*^+^ were introduced into the cells as described in Chikashige et al. (2006). GFP-tagged Meu14 or LifeAct was expressed from the *lys1^+^*-integrating plasmid.

### Live-cell imaging of sporulating cells

After overnight incubation on ME plates, cells were re-suspended in EMM-N+5S medium. To disperse sporulating cells, suspensions were subjected by brief sonication (Handy Sonic; Tomy Seiko, Tokyo, Japan); 20 µl of the cell suspension was then dropped onto lectin (0.2 mg/ml; Sigma-Aldrich, Tokyo, Japan)-coated 35-mm glass-bottomed culture dishes (MatTek, Ashland, MA, USA) to immobilize cells (Asakawa and Hiraoka, 2009). For imaging Latrunculin A-treated cells, Latrunculin A (Thermo Fisher Scientific, Tokyo, Japan) was added at a final concentration of 1 µM prior to cell immobilization. Cells undergoing sporulation were selected for live-cell imaging.

A DeltaVision microscope equipped with a CoolSNAP HQ^2^ charge-coupled device (GE Healthcare, Tokyo, Japan) was used for image acquisition. Optical section images were acquired at 0.5-µm focus intervals using an oil-immersion 60× objective lens (PlanApoN60x OSC, NA1.4; Olympus, Tokyo, Japan). Images were processed using the de-noising algorithm (Boulanger et al., 2009) and by constrained iterative deconvolution (Agard et al., 1989).

### EM imaging

Cells were induced to sporulate on ME plates overnight, and aliquoted in monolayers on lectin-coated glass-bottomed culture dishes with addressing grids (grid size 50 µm; ibid, Bremen, Germany). Cells were fixed with 2% glutaraldehyde (Polysciences, Inc., Warrington, PA, USA) in 0.1 M phosphate buffer (pH 7.2) for 2 h at 4°C. Optical section images (0.2-µm intervals) of a cell of interest were obtained using the Olympus objective lens, as described above. EM observation was performed as described previously (Asakawa et al., 2010). Briefly, cells were post-fixed with a 1.2% KMnO_4_ solution overnight at 4°C and embedded in Epon812. The epoxy block containing the same cells observed by fluorescence microscopy was trimmed according to the location on the coverslip. Serial sections with 80-nm thickness were stained with 4% uranyl acetate and a commercial ready-to-use solution of lead citrate (Sigma-Aldrich, St. Louis, MO, USA), and analyzed using a JEM1400 transmission electron microscope (JEOL, Tokyo, Japan). Adobe Photoshop CS4 (ver.11.0.1) was used for image processing.
